# The relationship between target joints and direct resource use in severe haemophilia

**DOI:** 10.1186/s13561-018-0185-7

**Published:** 2018-01-16

**Authors:** Jamie O’Hara, Shaun Walsh, Charlotte Camp, Giuseppe Mazza, Liz Carroll, Christina Hoxer, Lars Wilkinson

**Affiliations:** 1 Faculty of Health and Social Care, University of Chester, Chester, UK; 2 HCD Economics, The Innovation Centre, Daresbury, WA4 4FS UK; 3 UCL Institute for Liver and Digestive Health, Royal Free Hospital, University College London, London, UK; 4 The Haemophilia Society, London, UK; 5 Novo Nordisk A/S, Vandtårnsvej 114, -2860 Søborg, DK Denmark

**Keywords:** Haemophilia, Cost of illness, Target joints, Burden of disease, Arthropathy, Synovitis

## Abstract

**Objectives:**

Target joints are a common complication of severe haemophilia. While factor replacement therapy constitutes the majority of costs in haemophilia, the relationship between target joints and non drug-related direct costs (NDDCs) has not been studied.

**Methods:**

Data on haemophilia patients without inhibitors was drawn from the ‘Cost of Haemophilia across Europe – a Socioeconomic Survey’ (CHESS) study, a cost assessment in severe haemophilia A and B across five European countries (France, Germany, Italy, Spain, and the United Kingdom) in which 139 haemophilia specialists provided demographic and clinical information for 1285 adult patients. NDDCs were calculated using publicly available cost data, including 12-month ambulatory and secondary care activity: haematologist and other specialist consultant consultations, medical tests and examinations, bleed-related hospital admissions, and payments to professional care providers. A generalized linear model was developed to investigate the relationship between NDDCs and target joints (areas of chronic synovitis), adjusted for patient covariates.

**Results:**

Five hundred and thirteen patients (42% of the sample) had no diagnosed target joints; a total of 1376 target joints (range 1–10) were recorded in the remaining 714 patients. Mean adjusted NDDCs for persons with no target joints were EUR 3134 (standard error (SE) EUR 158); for persons with one or more target joints, mean adjusted NDDCs were EUR 3913 (SE EUR 157; average mean effect EUR 779; *p* < 0.001).

**Conclusions:**

Our analysis suggests that the presence of one or more target joints has a significant impact on NDDCs for patients with severe haemophilia, ceteris paribus. Prevention and management of target joints should be an important consideration of managing haemophilia patients.

## Background

Haemophilia is an inherited, lifelong bleeding disorder characterised by prolonged traumatic or spontaneous bleeding due to a lack of clotting factor in the body. Haemophilia is a recessive X-linked disorder and primarily affects males; symptoms are present from infancy [1]. The two most common forms of the condition are Haemophilia A (Factor VIII deficiency) and Haemophilia B (Factor IX deficiency). Global incidence of haemophilia A is approximately 1 in every 5000 male births; haemophilia B is approximately six times rarer than haemophilia A [2].

Bleed events may be musculoskeletal or mucosal in nature but are most commonly observed in the joints of the body. In the absence of preventative ‘prophylaxis’ factor replacement therapy, most persons with severe haemophilia (<1% of normal factor level) will develop a first haemarthrosis between the ages of 1 and 5 years. Approximately four-fifths of bleed events occur in the knees, elbows, and ankles; arthroses in the hip, shoulder, carpus, or small hand or foot joints are less frequently observed [3].

Repeat intra-articular bleed events within a short timeframe (3–6 months) are associated with chronic synovial inflammation and in the longer term induce haemophilic arthropathy [4]. Such joints, known as target joints, exhibit continuous swelling and reduced range of motion; repeat acute and subacute haemarthoses lead to irreversible degradation of the joint, resulting in chronic pain and poor physical function and requiring orthopaedic intervention, ranging from removal of the synovium to replacement or fusion of the joint [5]. Bleed frequency, as well as age, body mass index, and inhibitor formation are known drivers of joint disease and functional limitation in persons with severe haemophilia [6–8]. The economic burden of frequent hospitalisations and palliative joint surgeries is reinforced by the psychosocial impact of chronic pain and disability, including limited employment opportunities, decreased social participation, and poor mental health [9, 10].

Prophylaxis regimens initiated at a young age (≤4 years of age) are shown to reduce bleed frequency and joint deterioration later into adulthood [5], and are therefore considered the benchmark in care for severe haemophilia [2]. However, introduction of universal prophylaxis has been protracted in many developed countries, due to a lack of evidence regarding clinical benefits of prophylaxis initiation later into adolescence and adulthood, as well as the substantial per-capita costs associated with replacement therapy [11–13]. Prophylaxis replacement therapy has recently undergone tentative economic evaluation by several European organisations. There is a need for greater clarity regarding the economic impact of care for persons with severe haemophilia in Europe, specifically regarding the cost of management of individuals with musculoskeletal complications, and in particular those patients receiving suboptimal therapy protocols.

The objective of this paper is to explore the relationship between target joints and direct medical costs for persons with severe haemophilia, and the extent to which health resource utilisation and direct medical costs (excluding replacement therapy) in severe haemophilia are driven by long-term clinical complications of the disease. While this topic has been explored to some detail within single-country studies [12, 14], this is the first to take a universal methodology across several European countries in assessing resource use and cost burden among persons with severe haemophilia.

## Methods

### Data source

Resource and cost data were gathered as part of the “Cost of Haemophilia across Europe – a Socioeconomic Survey (CHESS)”, a prospective observational study in severe haemophilia A and B across five European countries (France, Germany, Italy, Spain, and the United Kingdom) undertaken in 2015 [15]. One hundred and thirty-nine haemophilia specialists provided demographic and clinical information for 1285 adults (≥18 years) via a web-based survey. A corresponding questionnaire covering indirect costs and health-related quality of life (HRQOL) measures was completed by patients.

Non drug-related direct costs (NDDCs) were an amalgam of 12-month ambulatory and secondary care costs gathered within the CHESS study, specifically incorporating: haematologist and other specialist consultant consultations, medical tests and examinations, surgeries relating to joint damage, bleed-related hospital admissions, and payments to professional care providers [15]. A unit cost database was developed for each country using publicly available information. A breakdown of individual cost elements of NDDCs is presented in Table 1.

**Table 1:** National costs for CHESS resource units

Resource item	Baseline unit price (EUR)				
	France^a^	Germany^b^	Italy^c^	Spain^d^	UK^e^
Ambulatory care					
Haematologist visit (per visit)	25.99–45.99	20.88	27.32–23.17	65.69–113.54	124.71–228.57
Nurse visit (per visit)	81.74	34.28–38.42	15.11	20.92–37.46	19.36
Other specialist visit (per visit)	14.99–45.99	7.30–228.88	18.21–27.32	16.42–160	65.91–612.03
Blood test (per test)	1.89–53.96	0.50–112.50	2.11–17.22	4.78–98.37	4.29–7.67
Other test/examination (per test)	10.79–69.00	5.50–124.60	2.19–134.27	7.49–249.21	1.69–228.24
Hospitalisation					
Target joint surgery† (per surgery)	28.81–534.40	12.02–1719.43	33.48–1032.91	169.75–2156.33	1161.93–8397.52
Bleed event: ward stay (per day)	290.85	514.29	265	708.71	562.88
Bleed event: ICU stay (per day)	1174.60	1265	366	1559.24	1056.82
Professional caregiver (per hour)	8.30	27.43	7.39	13.66	24.56

Note. Ranges presented where more than one price is possible; ICU: intensive care unit; IU: International Units

†Arthrocentesis, arthrodesis, arthroplasty, arthroscopy, synovectomy

^a^Sources: Ameli, sante.gouv, ViDAL.fr, Catalogue Commun des actes médicaux

^b^Sources: Kbv.de, meinpharmaversand.de, Einheitlicher Bewertungsmaßstab, rote-liste service

^c^Sources: AIFA, agenziafarmaco.gov

^d^Sources: Oblikue e-salud, Agencia Española de Medicamentos y Productos Sanitarios

^e^Sources: National Schedule of Reference Costs, Electronic Medicines Compendium

Study exclusion criteria was limited to patients diagnosed with an inhibitor at the time of study capture (*n* = 52), due to a differing risk profile for bleeds and subsequent target joint development among these patients, and a higher utilisation of medical resources [16, 17].

### Target joints

A ‘target joint’ as defined in the CHESS study encompasses any joint with known chronic synovitis; in contrast to previous clinical studies [18], study investigators were given discretion as to how this may be further defined with respect to bleed frequency and period of observation. In order to explore the differential impact of costs associated with lower and upper body joint deterioration, target joints were categorised into two groups based on their location. ‘Upper body’ target joints were those in the shoulders, elbows, wrists, neck, and spine; ‘lower body’ target joints consisted of hips, knees, and ankles. The target joint variable was assessed in three ways: as a binary 0/1 variable; as a binary 0/1 variable split into upper and lower body joints; and as a discrete variable.

### Statistical analysis

Demographic and resource use data were compared between the sample of patients with no reported target joints and those with one or more reported target joints. Means were used to describe continuous variables; categorical variables are described as frequencies and proportions. Standard t-tests were conducted in order to test for between-group differences.

The marginal effect of the presence of one or more target joints on NDDCs was assessed using a generalized linear model (GLM). Medical cost data is often positively skewed with a large volume of zero values (i.e. no medical costs) and a long ‘tail’ from a select group of costly ‘outlier’ patients. The GLM is an extension of the linear regression framework (Eq. 1) suitable for nonparametric dependent variables [19, 20]. The GLM requires a link function relating the conditional mean to the covariates, and a distribution ‘family’ to specify the relationship between the variance and the mean [21]. The log-link function (Eq. 2) in combination with a gamma distribution (Eq. 3) is frequently used to estimate medical costs and was employed for this analysis [21]. A confirmatory analysis of the family and link functions was conducted using the modified Park test [20, 21].

1 $$ {NDDCs}_{12 mth}=\alpha +{\beta}_1\left(t\mathit{\arg} et\; jo\mathit{\operatorname{int}}s\right)+{\beta}_2{x}_1+\cdots +{\beta}_n{x}_n $$

$$ Where\;i=1,\dots, n $$

2 $$ E\left[y\left|x\right.\right]=f\left({x}^{\hbox{'}}\beta \right)=\exp\;\left({x}^{\hbox{'}}\beta \right)\; In\;\left(E\left[y\left|x\right.\right]\right)={x}^{\hbox{'}}\beta $$

3 $$ y\sim Var\left(y\left|x\right.\right)\approx {\left(E\left[y\left|x\right.\right]\right)}^{\lambda } $$

A univariate estimate of the relationship between target joint status and NDDCs was first modelled (Model 1), followed by a multivariate estimate using country of residence, patient age, use of prophylaxis therapy regimen at the time of study capture, and number of haemophilia-related hospital admissions in the preceding 12 months as additional model covariates, added using a stepwise inclusion method (Model 2). Results are presented as average mean effects (AME). All statistical analysis was conducted using Stata 13 [22].

## Results

### Patient characteristics

The average age of study patients was 36 years old; the majority of patients in the study were receiving treatment prophylactically (*n* = 708; 57.7%) (Table 2). A total of 1376 target joints were recorded across the study population (mean 1.2 target joints; SD 1.37; range 0–10). Seven hundred and fourteen patients (58.2%) were reported diagnosed with one or more target joints. Target joints exclusively in the lower body were most commonly reported (*n* = 371 patients (52.3%)). More than four in ten patients with a reported target joint had undergone one or more surgeries on a target joint in the preceding 12 months, with joint aspiration (arthrocentesis) the most common procedure (200 patients, 28% of the target joint cohort).

**Table 2:** Patient characteristics (*N* = 1227)

Age (mean ± SD)	35.7 ± 14.6
Subtype (%)	
Haemophilia A	949 (77.3%)
Haemophilia B	278 (22.7%)
Country (%)	
UK	306 (24.9%)
Italy	271 (22.1%)
France	254 (20.7%)
Spain	206 (16.8%)
Germany	190 (15.5%)
Treatment strategy (%)	
On-demand	519 (42.3%)
Prophylaxis	708 (57.7%)
Target joints (mean ± SD)	1.1 ± 1.4
Number of target joints (patient n, %)	
Zero	518 (41.2%)
One	334 (46.4%)
Two	241 (34.0%)
Three or more	139 (19.6%)
Location of target joints (patient n, % of target joint cohort)	
Exclusively lower body	371 (52.3%)
Exclusively upper body	186 (26.2%)
Upper and lower body	152 (21.4%)
Surgeries to target joints in the previous 12 months (patient n, % of target joint cohort)	305 (42.7%)
Joint aspiration	200 (28.0%)
Endoscopic examination of joint	116 (16.2%)
Joint fusion	84 (11.8%)
Joint replacement	64 (9.0%)
Destruction/surgical removal of synovium	56 (7.8%)

*Note.* Values are means ± SD or numbers (%)

### Medical resource use

In all cases examined, patients with no target joints consumed less medical resources compared to patients with one or more target joints (Table 3). The largest between-group differences were reported for scheduled nurse consultations with 5.75 (SD 11.98) and 3.94 (SD 9.13) for the “has target joint” and “no target joint” groups respectively (*p* < 0.001). Physiotherapy visits were found to be lower in the no target joint group 0.89 (SD = 3.75) compared with patients with one or more target joints 3.14 (SD = 7.99) (*p* < 0.001). Patients with target joints attended a greater number of scheduled haemophilia consultations: 5.5 (SD = 4.36) and 4.7 (SD = 3.72) respectively (*p* = 0.001). The target joint group recorded 1.91 GP visits (SD = 3.78), with non-target joint patients reporting 1.35 visits (SD = 2.67; *p* = 0.003). Mean number of target joint surgeries in the affected group was 0.70. Rates of bleed-related hospital admissions were almost three times higher in the target joint cohort (mean 0.97 versus 0.36).

**Table 3:** 12-month resource utilisation (*N* = 1227)

	No target joint (*n* = 508)	1+ target joints (*n* = 714)	*P*-Value
Outpatient haematologist consultations			
Scheduled	4.71	5.48	0.001
Unscheduled	1.15	1.84	0.001
Outpatient specialist nurse consultations			
Scheduled	3.95	5.75	0.003
Unscheduled	1.13	1.93	<0.001
Outpatient consultations – other specialties			
General practice	1.35	1.91	<0.001
General surgery	0.08	0.21	<0.001
Pain management	0.11	0.68	<0.001
Physiotherapy	0.89	3.14	<0.001
Tests and examinations			
Urinalysis	1.07	1.85	<0.001
X-ray	0.61	1.24	<0.001
Computed tomography	0.25	0.48	<0.001
Magnetic resonance imaging	0.25	0.52	<0.001
Radiography	0.36	1.05	<0.001
Ultrasonography	0.41	1.00	<0.001
Coagulation tests	1.82	3.11	<0.001
Target joint surgeries	n/a	0.70	n/a
Bleed event-related hospital admissions	0.36	0.97	<0.001

### NDDCs

**Fig. 1:**
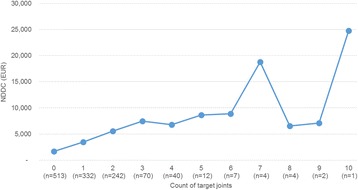
NDDCs by count of target joints (*N* = 1227)

Mean NDDCs were EUR 3641 (SD 6157); per-patient NDDCs in the presence of one or more target joints – regardless of number or location – was EUR 5046 (SD 7479) versus EUR 1684 for patients with no target joints. The number of target joints are positively correlated with NDDCs: individuals with one target joint reported mean NDDCs of EUR 3468 (SD 5595; *n* = 332) (Fig. 1); this increased to EUR 5585 (SD 7980; *n* = 242) for patients with two target joints; for those with three target joints mean NDDCs were EUR 7470 (SD 9396; *n* = 70).

**Fig. 2:**
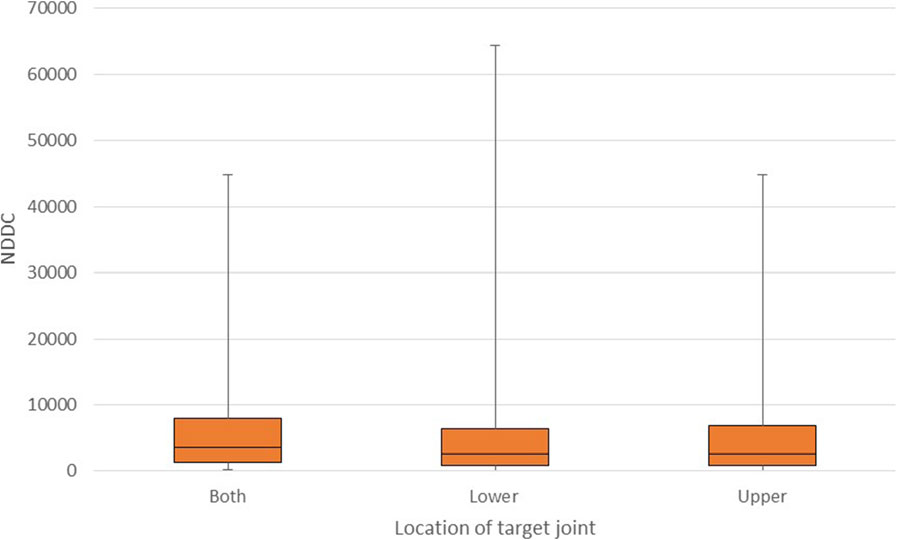
NDDCs by target joint location (*N* = 714)

Patients with at least one target joint in the upper body recorded mean NDDCs of EUR 5610 (SD 7861) (Fig. 2); patients with at least one lower body target joint reported mean NDDCs of EUR 5186 (SD 7594). The highest NDDCs were recorded among patients with both lower and upper body target joints (mean EUR 6696; SD 8461).

### Multivariate analysis

The results of the multivariate analysis are presented in Table 4. A significant difference in costs was observed between the target joint and non-target joint cohorts: mean adjusted NDDCs for the non-target joint cohort were EUR 3134 (SE 158); for the target joint cohort, mean adjusted NDDCs were EUR 3913 (SE 157; AME EUR 779; *p* < 0.001). The mean average marginal effect (AME) of one or more upper body target joints was EUR 2646 (standard error (SE) 454); AME was EUR 2626 (SE 367) for individuals with one or more lower body target joints. When the analysis examined the impact of the location of the target joint, the AME for a lower body target joint was greater, at EUR 655 (SE 143). The AME for a patient with an upper body target joint was EUR 624 (SE 191).

**Table 4:** Multivariate Gamma regression analyses of NDDCs (*N* = 1227)

	Model 1	Model 2
Upper body target joint	2645.63 (454.07)	623.51 (191.13)**
Lower body target joint	2626.32 (367.36)	665.00 (143.57)**
Country^a^		
Germany		−711.36 (131.30)**
Italy		−562.68 (134.09)**
Spain		569.75 (254.91)*
UK		439.2861 (212.43)*
Age		13.52 (3.48)**
Haemophilia hospitalisation		2681.52 (220.15)**
On-demand treatment strategy		140.21 (59.65)*
	AIC = 18.13 BIC = −6939.61	AIC = 17.59 BIC = −7600.63

*Note*. Values are average marginal effects (AMEs). Standard error shown in brackets. *Denotes 95% significance. **Denotes 99% significance

^a^Base factor: France

Patient age was a negligible – albeit significant – driver of NDDCs: an additional year of life contributed just EUR 13.52 extra in costs (SD 3.48; *p* < 0.001). Somewhat unsurprisingly, an additional haemophilia-related hospitalisation was the most substantial contributor to NDDCs (AME 2681.52; SD 220.15; *p* < 0.001).

## Discussion

This study has sought to quantify the economic burden associated with management and alleviation of joint-related complications in severe haemophilia. NDDCs represent a small proportion of total costs for persons with severe haemophilia (between 2% and 5% in most European studies) [14], and as a result their prioritisation for research has until now been limited. Nevertheless, it is critical to understand both physiological, psychosocial, and economic impacts of inadequate bleed control within haemophilia, and the longer-term repercussions of joint deterioration associated with suboptimal therapy and patient management. The results of this analysis suggest that cost drivers are not limited to surgical interventions, but in fact encompass more intensive ambulatory and outpatient care, including a higher volume of specialist visits, tests, and examinations among persons with severe haemophilia exhibiting chronic joint inflammation.

The CHESS study was a cross-sectional survey of clinicians and persons with severe haemophilia across five European countries, which captured a large volume of clinical and demographic information about consulting patients. However, we are limited in our ability to explore the causal relationship between management of haemophilia in early life and subsequent outcomes. Further work is required to understand the long-term impact of switching from on-demand to prophylaxis in late childhood and adulthood, when joint deterioration may already be present. Presence of target joints in the CHESS patient group is significantly higher among those receiving prophylaxis; whilst initially counterintuitive, it points to a large number of patients moving away from on-demand regimens due to poor bleed control and joint damage. As shown in previous work, this is particularly pertinent for those patients over the age of 30 years for whom prophylaxis in early childhood was not widely available. While the outcomes observed in this study population are not necessarily translatable to children born with haemophilia in the current era, suggestions of reducing access to prophylaxis give rise to a need to highlight the economic burden arising from conservative therapy among persons with severe haemophilia.

A target joint in this analysis is defined by the presence of chronic synovitis, the physiological manifestation of frequent intra-articular bleed events. In the more oft-observed scenario, sufficient time between bleed events (~3–4 weeks) allows for gradual alleviation of swelling and restoration of joint motion, via intensive infusions of replacement therapy and regular physiotherapy. Frequent recurrence of bleeding, however, precludes the reinstatement of a baseline level of motion, strength, and physical appearance; inadequate resolution of trauma arising from such events can in turn exacerbate bleed frequency. This cyclical process results in the longer term in a state of chronic synovitis and progressive arthropathy, due to excessive volumes of synovium and blood retained within the joint space and gradual deterioration of the joint tissue [4].

Other definitions in literature focus instead on the frequency of bleed events over a short-term (≤6 months) period in order to propose a diagnosis of a target joint. The most recent International Society of Thrombosis and Hemostasis (ISTH) definition, for example, is one in which three or more spontaneous bleeds into a single joint occur within a consecutive 6-month period [23]. In the case of the ISTH guidelines, however, the joint ceases to be a target joint when there have been less than two bleeds into the joint within 12 consecutive months. While alternative definitions allow for more or less frequent bleeds and shorter or longer observation periods [24, 25], their commonality is the use of short-term bleed rates as a measure, and an observable follow-up period within which a target joint may no longer be defined as such.

In combination, these rules present a definition that is well-suited to clinical trials with finite follow-up periods and observable improvements in outcomes, but which lacks the consideration of the long-term complications associated with persistent bleed events. A recent paper published on behalf of the United Kingdom Haemophilia Centres Doctors Organisation (UKHCDO) highlights the long term changes effected to the soft tissue of the joint as a result of frequent bleed events, and thus a need for continuous, targeted monitoring of the afflicted joint beyond the period in which synovitis is observed [26]. Our choice of definition, therefore, encompasses an assumption of high bleed frequency via the identification of synovitis, as well as considering the long-term, irreversible changes to the joint tissue and structure that arise from repeat haemorrhage. We acknowledge, however, that there is substantial overlap between definitions.

Regardless of nuances in the definition of a target joint, the results presented in this study suggest that chronic synovitis in severe haemophilia is associated with greater intensity of patient management, resulting in higher levels of health resource use and direct medical costs. Approaches to minimising the long-term risk of joint damage and deterioration among these patients – beginning at a young age with proactive therapy protocols to minimise bleed frequency and severity – will serve to reduce future burdens on hospital systems and are a justification for continued access to preventative therapy protocols. Further studies should seek to incorporate the health-related quality of life impact of bleed events and joint disease, and hence to quantify the cost effectiveness of current therapy protocols among severe persons with haemophilia.

## Conclusion

While non drug-related direct medical costs in severe haemophilia are small in relation to the costs of replacement therapy, our analysis demonstrates that the majority of individuals with this disease experience medical complications requiring substantial follow-up in the hospital setting. Further, the presence of one or more target joint can have a major impact on medical resource utilisation and subsequent costs for patients with severe haemophilia.

## Abbreviations

AME: average mean effect
CHESS: ‘The Cost of Haemophilia – a Socioeconomic Survey’
EUR: Euro
GLM: generalized linear model
HRQOL: health-related quality of life
ICU: intensive care unit
ISTH: International Society on Thrombosis and Haemostasis
IU: international units
PWH: people with haemophilia
SD: standard deviation
SE: standard error

## References

[1] Liras A, Segovia C, Gabán AS (2012). Advanced therapies for the treatment of hemophilia: future perspectives. Orphanet J. Rare Dis..

[2] Srivastava A, Brewer AK, Mauser-Bunschoten EP, Key NS, Kitchen S, Llinas A (2013). WFH guidelines: guidelines for the management of hemophilia. Haemophilia.

[3] Lobet S, Hermans C, Lambert C (2014). Optimal management of hemophilic arthropathy and hematomas. J Blood Med Dove Press.

[4] Mulder K, Llinás A (2004). The target joint. Haemophilia.

[5] Manco-Johnson MJ, Soucie JM, Gill JC (2017). Prophylaxis usage, bleeding rates and joint outcomes of hemophilia 1999 - 2010: a surveillance project. Blood.

[6] Monahan PE, Baker JR, Riske B, Soucie JM (2011). Physical functioning in boys with hemophilia in the U.S. Am J Prev Med.

[7] Bladen M, Main E, Hubert N, Koutoumanou E, Liesner R, Khair K (2013). Factors affecting the Haemophilia joint health score in children with severe haemophilia. Haemophilia.

[8] Soucie JM, Wang C, Siddiqi A, Kulkarni R, Recht M, Konkle BA (2011). The longitudinal effect of body adiposity on joint mobility in young males with Haemophilia a. Haemophilia.

[9] Iannone M, Pennick L, Tom A, Cui H, Gilbert M, Weihs K (2012). Prevalence of depression in adults with haemophilia. Haemophilia.

[10] Cassis FRMY, Querol F, Forsyth A, Iorio A, HERO International Advisory (2012). Board. Psychosocial aspects of haemophilia: a systematic review of methodologies and findings. Haemophilia.

[11] World Federation of Hemophilia. Frequently asked questions about hemophilia [Internet]. 2012 [cited 2015 Jul 31]. Available from: http://www.wfh.org/en/page.aspx?pid=637#Life_expectancy.

[12] Johnson KA, Zhou Z-Y (2011). Costs of care in hemophilia and possible implications of health care reform. Hematology am. Soc. Hematol. Educ. Program.

[13] Henrard S, Hermans C, Devleesschauwer B, Speybroeck N (2012). Oral presentations: abstracts. Eur J Public Health O.

[14] Kodra Y, Cavazza M, Schieppati A, De Santis M, Armeni P, Arcieri R, et al. The social burden and quality of life of patients with haemophilia in Italy. Blood Transfus. SIMTI Servizi; 2014;12 Suppl 3:s567–s575. 10.2450/2014.0042-14s PMC4044804 24922297

[15] O’Hara J, Hughes D, Camp C, Burke T, Carroll L (2017). Diego D-AG. The cost of severe haemophilia in Europe: the CHESS study. Orphanet J Rare Dis.

[16] Bohn RLRL, Aledort LM, Putnam K, Ewenstein B, Mogun H, Avorn JJ (2004). The economic impact of factor VIII inhibitors in patients with haemophilia. Haemophilia.

[17] Knight C (2005). Health economics of treating haemophilia a with inhibitors. Haemophilia.

[18] Konkle BA, Ebbesen LS, Erhardtsen E, Bianco RP, Lissitchkov T, Rusen L (2007). Randomized, prospective clinical trial of recombinant factor VIIa for secondary prophylaxis in hemophilia patients with inhibitors. J Thromb Haemost Blackwell Publishing Ltd.

[19] Glick HA, Doshi JA, Sonnad SS, Polsky D. Economic Evaluation in Clinical Trials. Oxford University Press; 2014.

[20] Mihaylova B, Briggs A, O’Hagan A, Thompson SG (2011). Review of statistical methods for analysing healthcare resources and costs. Health Econ.

[21] Coughlan D, Yeh ST, Neill CO, Frick KD (2014). Evaluating direct medical expenditures estimation methods of adults using the medical expenditure panel survey : an example focusing on head and neck cancer. Value Heal Elsevier.

[22] Stata Statistical Software. College Station: StataCorp; 2013.

[23] Blanchette VS, Key NS, Ljung LR, Manco-Johnson MJ, van den Berg HM, Srivastava A (2014). Definitions in hemophilia: communication from the SSC of the ISTH. J Thromb Haemost.

[24] Price VE, Hawes SA, Chan AK (2007). A practical approach to hemophilia care in children. Paediatr Child Heal.

[25] Donadel-Claeyssens S (2006). European Paediatric network for Haemophilia management. Current co-ordinated activities of the PEDNET (European Paediatric network for Haemophilia management). Haemophilia.

[26] Hanley J, McKernan A, Creagh MD, Classey S, McLaughlin P, Goddard N (2017). Guidelines for the management of acute joint bleeds and chronic synovitis in haemophilia: a United Kingdom Haemophilia Centre doctors’ organisation (UKHCDO) guideline. Haemophilia.

